# (*R*)-*N*-(Ferrocenylmeth­yl)-1-hy­droxy-3-phenyl­propan-2-aminium (*E*)-but-2-enoate

**DOI:** 10.1107/S1600536811044096

**Published:** 2011-10-29

**Authors:** Petr Štěpnička, Ivana Císařová

**Affiliations:** a Department of Inorganic Chemistry, Faculty of Science, Charles University in Prague, Hlavova 2030, 12840 Prague 2, Czech Republic

## Abstract

The crystal structure of the title salt, [Fe(C_5_H_5_)(C_15_H_19_NO)](C_4_H_5_O_2_), consists of discrete ammonium and carboxyl­ate ions, which associate into infinite chains parallel to [100] by means of N—H⋯O and O—H⋯O inter­actions. These chains are further cross-linked into a three-dimensional network by additional C—H⋯O contacts and by offset π–π stacking inter­actions of inversion-related aromatic rings [centroid–centroid distance = 3.7040 (14) Å]. The mol­ecular parameters of the ionic components are in no way unexpected, the geometry of the ammonium cation being similar to that found in other structurally characterized salts obtained from *N*-ferrocenylmethyl *β*-amino­alcohols. The (*E*)-but-2-enoate anion consists of two approximately planar subunits, *viz* the delocalized carboxyl­ate unit and the butenyl group (the latter being planar within *ca.* 0.002 Å), which are mutually rotated by 30.3 (4)°.

## Related literature

For crystal structures of *N*-ferrocenylmethyl *β*-amino­alcohols and their salts, see: Štěpnička *et al.* (2004 ▶, 2008**a* ▶,b* ▶). For the preparation of a simple *N*-ferrocenylmethyl *β*-amino­alcohol, FcCH_2_NHCH_2_CH_2_OH (Fc = ferrocen­yl), see: Hess *et al.* (1999 ▶). For an overview of organometallic crystal engineering, see: Braga *et al.* (2008 ▶) and references cited therein.
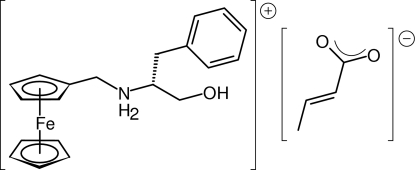


## Experimental

### 

#### Crystal data

[Fe(C_5_H_5_)(C_15_H_19_NO)](C_4_H_5_O_2_)
*M* *_r_* = 435.33
Monoclinic, 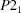

*a* = 5.9730 (2) Å
*b* = 15.3905 (3) Å
*c* = 11.7713 (4) Å
β = 100.4986 (13)°
*V* = 1063.99 (6) Å^3^
*Z* = 2
Mo *K*α radiation
μ = 0.73 mm^−1^
*T* = 150 K
0.33 × 0.12 × 0.10 mm

#### Data collection

Nonius KappaCCD diffractometer
15916 measured reflections
4864 independent reflections
4511 reflections with *I* > 2σ(*I*)
*R*_int_ = 0.043

#### Refinement

*R*[*F* ^2^ > 2σ(*F* ^2^)] = 0.030
*wR*(*F* ^2^) = 0.067
*S* = 1.05
4864 reflections
265 parameters
1 restraint
H-atom parameters constrained
Δρ_max_ = 0.36 e Å^−3^
Δρ_min_ = −0.27 e Å^−3^
Absolute structure: Flack (1983 ▶), 2329 Friedel pairs
Flack parameter: −0.016 (12)

### 

Data collection: *COLLECT* (Nonius, 2000 ▶); cell refinement: *SCALEPACK* (Otwinowski & Minor, 1997 ▶); data reduction: *DENZO* (Otwinowski & Minor, 1997 ▶) and *SCALEPACK*; program(s) used to solve structure: *SIR97* (Altomare *et al.*, 1999 ▶); program(s) used to refine structure: *SHELXL97* (Sheldrick, 2008 ▶); molecular graphics: *PLATON* (Spek, 2009 ▶); software used to prepare material for publication: *SHELXL97* and *PLATON*.

## Supplementary Material

Crystal structure: contains datablock(s) I, global. DOI: 10.1107/S1600536811044096/bh2388sup1.cif

Structure factors: contains datablock(s) I. DOI: 10.1107/S1600536811044096/bh2388Isup2.hkl

Additional supplementary materials:  crystallographic information; 3D view; checkCIF report

## Figures and Tables

**Table 1:** Hydrogen-bond geometry (Å, °)

*D*—H⋯*A*	*D*—H	H⋯*A*	*D*⋯*A*	*D*—H⋯*A*
N1—H91⋯O2^i^	0.95	1.74	2.685 (2)	173
N1—H92⋯O3	0.82	1.94	2.747 (2)	170
O1—H93⋯O3^i^	0.87	1.85	2.712 (2)	172
C16—H16⋯O1^ii^	0.93	2.56	3.447 (3)	159
C18—H18⋯O2^iii^	0.93	2.58	3.435 (3)	154

Symmetry codes: (i) 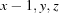; (ii) 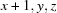; (iii) 
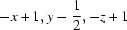
.

**Fig. 1.**
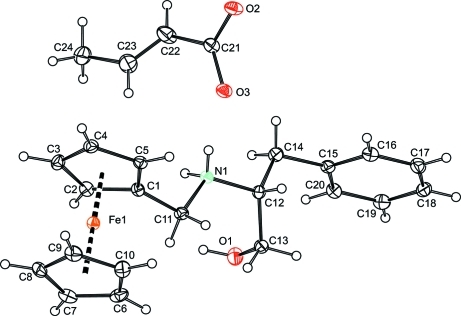
View of the structure of the title compound showing displacement ellipsoids for non-H atoms at the 30% probability level.

**Fig. 2.**
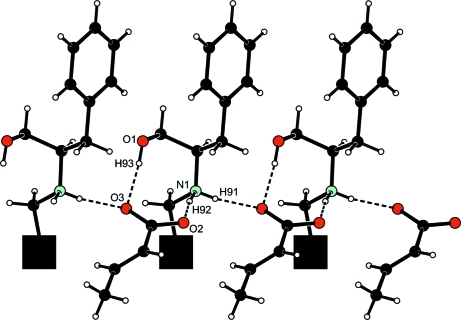
Section of the infinite H-bonded chains in the crystal structure of the title compound. The bulky ferrocenyl moieties were replaced with filled squares for clarity.

**Fig. 3.**
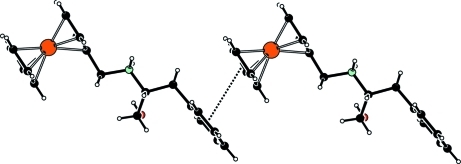
π···π Stacking interactions of the aromatic rings in the structure of the title compound (indicated by a dotted line). The molecules depicted relate by unit-cell translation in the direction of the c-axis.

## supplementary crystallographic information

### Comment

With our recent work (Štěpnička *et al.*, 2004 and 2008**a*,b*), we demonstrated that *N*-ferrocenylmethyl *β*-aminoalcohols with general formula FcCH_2_NHC*R*^1^*R*^2^C*R*^3^*R*^4^OH (Fc = ferrocenyl) are potentially useful building blocks for organometallic crystal engineering (Braga *et al.*, 2008 and references therein). These compounds possess a balanced number of conventional H-bond donors and acceptors (OH and NH) in their native form. When reacted with protonic acids, they are readily converted to the corresponding ammonium salts, the crystal assembly of which receives additional support from charge interactions. This contribution reports the crystal structure of a salt obtained from a chiral aminoalcohol, namely (*R*)-2-[(ferrocenylmethyl)azonia]-3-phenylpropan-1-ol (*E*)-but-2-enoate.

Several crystals of the title compound were isolated unexpectedly during attempted crystallization of (*R*)-2-[(ferrocenylmethyl)amino]-3-phenylpropan-1-ol from ethyl acetate/hexane, apparently resulting from the reaction of the free amine with (*E*)-but-2-enoic acid present as a trace impurity in reagent grade ethyl acetate. A view of the molecular structure is presented in Fig. 1.

The geometry of the cation is rather unexceptional, and compares well with those reported earlier for salts obtained from FcCH_2_CMe_2_CH_2_OH and similar *N*-ferrocenylmethyl *β*-aminoalcohols (Štěpnička *et al.*, 2004 and 2008**a*,b*). The ferrocenylmethyl and benzyl group attached to the 'central' N1—C12 bond assume an *anticlinal* eclipsed conformation (*cf*. torsion angle C11—N1—C12—C14) while their aromatic rings, C(1–5) and C(15–20), are nearly parallel [dihedral angle 7.75 (12)°] but mutually offset. The CH_2_OH pendant group is appended in a *gauche* position (*cf*. torsion angle C11—N1—C12—C13). Atom C13 is directed towards the ferrocenyl group and the C13—O1 bond extends away from the N1—C12 bond (*cf*. torsion angle N1—C12—C13—O1).

The ferrocenyl group shows negligible tilting [dihedral angle of the least-squares cyclopentadienyl planes is 1.63 (13)°] and similar Fe1—ring centroid distances [1.6461 (10) Å and 1.6565 (10) Å for the rings C(1–5) and C(6–10), respectively]. Although the Fe—C distances between Fe1 and individual carbon atoms in the substituted cyclopentadienyl ring C(1–5) differ by less than 0.03 Å [2.0271 (16)–2.054 (2) Å], there is a clear trend with the Fe—C distance gradually increasing from the C*_ipso_* to the opposite edge of the five-membered ring (*ipso* < *α* < *β*). The Fe—C distances observed for the unsubstituted cyclopentadienyl ring vary significantly less [2.040 (2)–2.058 (2) Å].

The C═C double bond within the (*E*)-but-2-enoate anion has an almost ideal *trans* configuration with the torsion angle C21—C22—C23—C24 of -179.6 (3)°, which renders the whole CH_3_—CH═CH—C group nearly perfectly planar (within *ca*. 0.002 Å). The terminal carboxyl group (C21, O2, O3) is symmetrically rotated from the plane of the CH_3_—CH═ CH—C moiety by as much as 30.3 (4)° and shows a delocalized character. The individual C—O distances differ by only *ca*. 0.02 Å and even this relatively small difference may come mainly from crystal packing effects as the longer C—O distance is associated with O3 acting as a double H-bond acceptor, while the shorter one involves O2 atom for which only one strong H-bond was detected.

The ions constituting the crystal of the title compound assemble by means of H-bonds between OH and NH groups and carboxylate oxygen atoms to form infinite chains in the [1 0 0] direction (Fig. 2). Distances between the H-bond donors and acceptors are close to 2.7 Å while the H-bond angles fall into the range 170–173° (Table 2). In addition, these conventional H-bonds are supported by the softer C—H···O interactions formed by CH groups at the terminal phenyl ring and proximal oxygen atoms O1 and O2 and further by π···π stacking interactions between the unsubstituted cyclopentadienyl ring C(6–10) and phenyl ring in a molecule related by unit-cell translation along the *c*-axis (Fig. 3). Least-squares planes of the interacting aromatic rings make a dihedral angle of 6.22 (12)°, and the distance of their respective centroids is 3.7040 (14) Å.

### Experimental

(*R*)-2-[(Ferrocenylmethyl)amino]-3-phenylpropan-1-ol was prepared by an established two-step procedure (Hess *et al.*, 1999; Štěpnička *et al.* 2004 and 2008*b*) consisting of condensation of ferrocene carboxaldehyde with (*R*)-phenylalaninol and subsequent reduction of the intermediate Schiff base as follows.

Ferrocene carboxaldehyde (428 mg, 2.00 mmol) and (*R*)-2-amino-3-phenylpropan-1-ol (318 mg, 2.1 mmol) were dissolved in dry chloroform (20 ml). The resulting solution was refluxed under argon for 90 min and then evaporated under vacuum. The residue was immediately re-dissolved in dry methanol (20 ml) and the solution was cooled in ice. An amount of NaBH_4_ (380 mg, 10 mmol) was added over 30 min causing the colour of the reaction mixture to change from initial orange red to orange yellow. After the addition, the reaction mixture was stirred at 0 °C for 1 h and at room temperature for 90 min before being quenched by addition of 10% aqueous NaOH and extracted with dichloromethane (2×20 ml). The combined organic extracts were washed with brine (2×20 ml) and dried over MgSO_4_ overnight.

The drying agent was filtered off and the filtrate was evaporated under vacuum leaving a residue which was purified by column chromatography over silica gel. Elution with dichloromethane–methanol (10:1 *v*/*v*) led to the development of two yellow bands. The first one containing ferrocenylmethanol was discarded. The second one was collected and evaporated under vacuum to afford (*R*)-2-[(ferrocenylmethyl)amino]-3-phenylpropan-1-ol (371 mg, 53%) as an orange amorphous solid.

Characterization. ^1^H NMR (CDCl_3_): δ 2.31 (br s, 2 H, NH and OH), 2.78 (dd, ^2^*J*_HH_ = 13.7, ^3^*J*_HH_ = 6.7 Hz, 1 H, CH_2_Ph), 2.83 (dd, ^2^*J*_HH_ = 13.7, ^3^*J*_HH_ = 7.6 Hz, 1 H, CH_2_Ph), 3.01 (m, 1 H, CHN), 3.38 (dd, ^2^*J*_HH_ = 10.8, ^3^*J*_HH_ = 4.7 Hz, 1 H, CH_2_O), 3.42 and 3.53 (2×d, ^2^*J*_HH_ = 12.9 Hz, 1 H, *AB* spin system of FcCH_2_), 3.67 (dd, ^2^*J*_HH_ = 10.8, ^3^*J*_HH_ = 3.8 Hz, 1 H, CH_2_O), 3.99 (s, 5 H, C_5_H_5_), 4.06 (m, 1 H, C_5_H_4_), 4.08 (virtual t, 2 H, C_5_H_4_), 4.14 (m, 1 H, C_5_H_4_), 7.18–7.34 (m, 5 H, Ph).

^13^C{^1^H} NMR (CDCl_3_): δ 38.15 (CH_2_Ph), 46.01 (FcCH_2_), 59.65 (CHN), 62.29 (CH_2_O), 67.66 (2 C), 67.77 and 67.89 (CH of C_5_H_4_); 68.34 (C_5_H_5_), 86.61 (C*_ipso_* of C_5_H_4_), 126.61 (1 C), 128.70 (2 C) and 129.18 (2 C) (3×CH of Ph); 138.44 (C*_ipso_* of Ph).

IR (neat): ν/cm^-1^ 3296 br s, 3086 s, 3026 m, 2924 s, 2856 m, 1653 br m, 1603 w, 1495 s, 1454 very strong, 1412 m, 1352 m, 1329 m, 1232 m, 1105 very strong, 1041 very strong br, 1001 s, 818 br very strong, 746 very strong, 700 very strong, 484 very strong.

MS: *m*/*z* (relative abundance) 350 (9), 349 (38, *M*^+.^), 347 (3), 331 (3, [*M* – H_2_O]^+.^), 266 (2), 200 (16), 199 (100, [FcCH_2_]^+^), 197 (6), 186 (2, FcH^+.^), 148 (3), 147 (3), 138 (2), 121 (28, [C_5_H_5_Fe]^+^), 91 (11), 78 (4), 77 (3), 65 (4), 56 (11, Fe^+^).

Few crystals of the title compound have been obtained upon attempted recrystallization of (*R*)-2-[(ferrocenylmethyl)amino]-3-phenylpropan-1-ol from ethyl acetate/hexane, resulting by a reaction of this compound with tiny amounts of (*E*)-but-2-enoic acid present in the commercial solvent (Lach-Ner).

### Refinement

N– and O-bonded H atoms were identified on the difference electron maps and isotropically refined as riding atoms. The remaining H-atoms were included in their calculated positions and refined as riding atoms with *U*_iso_(H) assigned to a multiple of *U*_eq_(C) of their bonding carbon atoms.

### Crystal data

**Table:**

[Fe(C_5_H_5_)(C_15_H_19_NO)](C_4_H_5_O_2_)	*F*(000) = 460
*M**_r_* = 435.33	*D*_x_ = 1.359 Mg m^−^^3^
Monoclinic, *P*2_1_	Mo *K*α radiation, λ = 0.71073 Å
*a* = 5.9730 (2) Å	Cell parameters from 2535 reflections
*b* = 15.3905 (3) Å	θ = 1.0–27.5°
*c* = 11.7713 (4) Å	µ = 0.73 mm^−^^1^
β = 100.4986 (13)°	*T* = 150 K
*V* = 1063.99 (6) Å^3^	Block, yellow
*Z* = 2	0.33 × 0.12 × 0.10 mm

### Data collection

**Table:**

Nonius KappaCCD diffractometer	4511 reflections with *I* > 2σ(*I*)
Radiation source: fine-focus sealed tube	*R*_int_ = 0.043
horizontally mounted graphite crystal	θ_max_ = 27.5°, θ_min_ = 3.2°
Detector resolution: 9.091 pixels mm^-1^	*h* = −7→7
ω and φ scans to fill the Ewald sphere	*k* = −19→20
15916 measured reflections	*l* = −15→15
4864 independent reflections	

### Refinement

**Table:**

Refinement on *F*^2^	Secondary atom site location: difference Fourier map
Least-squares matrix: full	Hydrogen site location: difference Fourier map
*R*[*F*^2^ > 2σ(*F*^2^)] = 0.030	H-atom parameters constrained
*wR*(*F*^2^) = 0.067	*w* = 1/[σ^2^(*F*_o_^2^) + (0.023*P*)^2^ + 0.4582*P*] where *P* = (*F*_o_^2^ + 2*F*_c_^2^)/3
*S* = 1.05	(Δ/σ)_max_ = 0.001
4864 reflections	Δρ_max_ = 0.36 e Å^−^^3^
265 parameters	Δρ_min_ = −0.27 e Å^−^^3^
1 restraint	Absolute structure: Flack (1983), 2329 Friedel pairs
0 constraints	Flack parameter: −0.016 (12)
Primary atom site location: structure-invariant direct methods	

### Fractional atomic coordinates and isotropic or equivalent isotropic displacement parameters (Å^2^)

**Table:**

	*x*	*y*	*z*	*U*_iso_*/*U*_eq_	
Fe1	0.34726 (4)	0.852498 (19)	1.26047 (2)	0.01975 (7)	
N1	0.2364 (3)	0.79109 (11)	0.88685 (13)	0.0188 (3)	
H91	0.1215	0.8326	0.8611	0.025 (6)*	
H92	0.3616	0.8129	0.8869	0.045 (8)*	
O1	−0.2057 (3)	0.69386 (11)	0.78892 (14)	0.0335 (4)	
H93	−0.2303	0.7427	0.8211	0.034 (7)*	
O2	0.9374 (3)	0.91779 (11)	0.81113 (14)	0.0316 (4)	
O3	0.6734 (2)	0.84425 (12)	0.88161 (12)	0.0279 (3)	
C1	0.3185 (3)	0.84770 (18)	1.08632 (14)	0.0202 (3)	
C2	0.1952 (4)	0.92054 (14)	1.11836 (18)	0.0235 (4)	
H2	0.0400	0.9306	1.0956	0.028*	
C3	0.3518 (4)	0.97480 (13)	1.19111 (18)	0.0260 (5)	
H3	0.3165	1.0267	1.2243	0.031*	
C4	0.5715 (4)	0.93652 (14)	1.20475 (18)	0.0243 (4)	
H4	0.7044	0.9587	1.2487	0.029*	
C5	0.5524 (3)	0.85864 (18)	1.13951 (15)	0.0209 (4)	
H5	0.6712	0.8211	1.1324	0.025*	
C6	0.2180 (4)	0.73848 (15)	1.30972 (19)	0.0322 (5)	
H6	0.1563	0.6940	1.2603	0.039*	
C7	0.0964 (4)	0.80938 (16)	1.3456 (2)	0.0319 (5)	
H7	−0.0592	0.8194	1.3245	0.038*	
C8	0.2551 (4)	0.86256 (18)	1.41971 (17)	0.0302 (5)	
H8	0.2219	0.9138	1.4551	0.036*	
C9	0.4725 (4)	0.82358 (14)	1.42989 (18)	0.0297 (5)	
H9	0.6072	0.8445	1.4736	0.036*	
C10	0.4491 (4)	0.74712 (15)	1.36194 (19)	0.0301 (5)	
H10	0.5659	0.7091	1.3532	0.036*	
C11	0.2253 (4)	0.77346 (13)	1.01152 (17)	0.0220 (4)	
H11A	0.0684	0.7634	1.0190	0.026*	
H11B	0.3118	0.7214	1.0368	0.026*	
C12	0.2090 (4)	0.71216 (13)	0.80983 (17)	0.0211 (4)	
H12	0.3477	0.6771	0.8297	0.025*	
C13	0.0092 (4)	0.65595 (13)	0.82677 (19)	0.0242 (4)	
H13A	0.0162	0.6017	0.7857	0.029*	
H13B	0.0245	0.6423	0.9083	0.029*	
C14	0.1931 (4)	0.74385 (14)	0.68492 (18)	0.0255 (4)	
H14A	0.0653	0.7834	0.6659	0.031*	
H14B	0.3305	0.7756	0.6785	0.031*	
C15	0.1632 (4)	0.66929 (13)	0.59961 (17)	0.0220 (4)	
C16	0.3474 (4)	0.61840 (14)	0.58549 (18)	0.0261 (5)	
H16	0.4909	0.6307	0.6280	0.031*	
C17	0.3201 (4)	0.54932 (15)	0.5087 (2)	0.0307 (5)	
H17	0.4447	0.5151	0.5009	0.037*	
C18	0.1077 (5)	0.53108 (15)	0.4434 (2)	0.0304 (5)	
H18	0.0896	0.4851	0.3912	0.037*	
C19	−0.0769 (4)	0.58170 (15)	0.45646 (19)	0.0294 (5)	
H19	−0.2199	0.5700	0.4129	0.035*	
C20	−0.0487 (4)	0.65025 (15)	0.53490 (19)	0.0258 (5)	
H20	−0.1739	0.6837	0.5440	0.031*	
C21	0.7467 (4)	0.91227 (15)	0.83988 (18)	0.0259 (4)	
C22	0.6023 (5)	0.99310 (18)	0.8262 (2)	0.0466 (7)	
H22	0.6189	1.0309	0.7667	0.056*	
C23	0.4576 (5)	1.01401 (17)	0.8904 (2)	0.0474 (7)	
H23	0.4445	0.9754	0.9496	0.057*	
C24	0.3080 (5)	1.09256 (16)	0.8816 (3)	0.0422 (6)	
H24A	0.3545	1.1333	0.8287	0.063*	
H24B	0.3207	1.1190	0.9563	0.063*	
H24C	0.1528	1.0757	0.8541	0.063*	

### Atomic displacement parameters (Å^2^)

**Table:**

	*U*^11^	*U*^22^	*U*^33^	*U*^12^	*U*^13^	*U*^23^
Fe1	0.02130 (13)	0.02038 (12)	0.01725 (12)	0.00067 (14)	0.00270 (9)	0.00026 (14)
N1	0.0175 (9)	0.0220 (8)	0.0167 (8)	−0.0016 (7)	0.0030 (6)	−0.0008 (6)
O1	0.0226 (8)	0.0361 (9)	0.0408 (9)	−0.0039 (7)	0.0033 (7)	−0.0117 (7)
O2	0.0224 (8)	0.0355 (9)	0.0386 (9)	0.0020 (7)	0.0104 (7)	0.0121 (7)
O3	0.0221 (7)	0.0293 (8)	0.0337 (7)	0.0016 (8)	0.0089 (5)	0.0043 (8)
C1	0.0221 (8)	0.0229 (9)	0.0153 (7)	0.0016 (11)	0.0027 (6)	0.0010 (10)
C2	0.0229 (11)	0.0263 (10)	0.0206 (10)	0.0053 (8)	0.0020 (8)	0.0042 (8)
C3	0.0335 (12)	0.0196 (10)	0.0245 (11)	0.0028 (9)	0.0042 (9)	0.0014 (8)
C4	0.0244 (11)	0.0250 (11)	0.0235 (10)	−0.0048 (8)	0.0041 (8)	−0.0013 (8)
C5	0.0202 (9)	0.0226 (9)	0.0204 (8)	0.0010 (10)	0.0046 (7)	0.0029 (10)
C6	0.0433 (14)	0.0284 (11)	0.0238 (11)	−0.0115 (10)	0.0031 (10)	0.0039 (9)
C7	0.0259 (12)	0.0436 (13)	0.0277 (12)	−0.0029 (10)	0.0086 (9)	0.0068 (10)
C8	0.0413 (12)	0.0327 (13)	0.0195 (9)	0.0010 (11)	0.0131 (8)	0.0008 (10)
C9	0.0325 (12)	0.0380 (13)	0.0171 (10)	−0.0031 (9)	0.0005 (9)	0.0027 (8)
C10	0.0389 (13)	0.0278 (11)	0.0228 (11)	0.0075 (10)	0.0038 (9)	0.0069 (9)
C11	0.0266 (11)	0.0232 (10)	0.0162 (9)	−0.0033 (8)	0.0039 (8)	0.0018 (8)
C12	0.0226 (10)	0.0219 (10)	0.0187 (10)	−0.0024 (8)	0.0037 (8)	−0.0022 (8)
C13	0.0233 (11)	0.0255 (11)	0.0241 (10)	−0.0038 (8)	0.0051 (8)	−0.0030 (8)
C14	0.0299 (12)	0.0243 (10)	0.0217 (10)	−0.0019 (9)	0.0033 (9)	0.0004 (8)
C15	0.0273 (11)	0.0240 (10)	0.0147 (9)	−0.0031 (9)	0.0034 (8)	0.0020 (8)
C16	0.0236 (11)	0.0298 (11)	0.0242 (11)	0.0000 (9)	0.0023 (9)	0.0044 (9)
C17	0.0309 (13)	0.0296 (12)	0.0337 (12)	0.0046 (9)	0.0114 (10)	0.0034 (10)
C18	0.0430 (16)	0.0248 (12)	0.0244 (11)	−0.0063 (10)	0.0085 (10)	−0.0012 (9)
C19	0.0292 (13)	0.0349 (12)	0.0228 (11)	−0.0055 (10)	0.0013 (9)	0.0005 (9)
C20	0.0246 (12)	0.0304 (12)	0.0216 (11)	0.0020 (10)	0.0026 (9)	0.0014 (9)
C21	0.0241 (11)	0.0303 (11)	0.0234 (10)	0.0041 (9)	0.0043 (9)	0.0058 (9)
C22	0.0508 (17)	0.0441 (15)	0.0521 (16)	0.0222 (13)	0.0286 (13)	0.0257 (13)
C23	0.064 (2)	0.0370 (14)	0.0464 (16)	0.0139 (13)	0.0238 (14)	0.0131 (12)
C24	0.0431 (16)	0.0321 (13)	0.0530 (16)	0.0061 (11)	0.0133 (13)	0.0033 (12)

### Geometric parameters (Å, °)

**Table:**

Fe1—C1	2.0271 (17)	C9—H9	0.9300
Fe1—C10	2.040 (2)	C10—H10	0.9300
Fe1—C2	2.041 (2)	C11—H11A	0.9700
Fe1—C5	2.0430 (18)	C11—H11B	0.9700
Fe1—C6	2.043 (2)	C12—C13	1.516 (3)
Fe1—C9	2.047 (2)	C12—C14	1.536 (3)
Fe1—C8	2.0533 (19)	C12—H12	0.9800
Fe1—C4	2.053 (2)	C13—H13A	0.9700
Fe1—C3	2.054 (2)	C13—H13B	0.9700
Fe1—C7	2.059 (2)	C14—C15	1.514 (3)
N1—C11	1.506 (2)	C14—H14A	0.9700
N1—C12	1.507 (2)	C14—H14B	0.9700
N1—H91	0.9454	C15—C16	1.385 (3)
N1—H92	0.8195	C15—C20	1.385 (3)
O1—C13	1.407 (3)	C16—C17	1.386 (3)
O1—H93	0.8666	C16—H16	0.9300
C1—C2	1.429 (3)	C17—C18	1.387 (4)
C1—C5	1.433 (3)	C17—H17	0.9300
C1—C11	1.487 (3)	C18—C19	1.381 (3)
C2—C3	1.419 (3)	C18—H18	0.9300
C2—H2	0.9300	C19—C20	1.392 (3)
C3—C4	1.420 (3)	C19—H19	0.9300
C3—H3	0.9300	C20—H20	0.9300
C4—C5	1.417 (3)	O2—C21	1.249 (3)
C4—H4	0.9300	O3—C21	1.268 (3)
C5—H5	0.9300	C21—C22	1.505 (3)
C6—C10	1.411 (3)	C22—C23	1.288 (4)
C6—C7	1.417 (3)	C22—H22	0.9300
C6—H6	0.9300	C23—C24	1.496 (3)
C7—C8	1.424 (3)	C23—H23	0.9300
C7—H7	0.9300	C24—H24A	0.9600
C8—C9	1.415 (3)	C24—H24B	0.9600
C8—H8	0.9300	C24—H24C	0.9600
C9—C10	1.415 (3)		
			
C1—Fe1—C10	121.63 (10)	C10—C6—H6	125.9
C1—Fe1—C2	41.13 (9)	C7—C6—H6	125.9
C10—Fe1—C2	158.18 (9)	Fe1—C6—H6	125.6
C1—Fe1—C5	41.24 (7)	C6—C7—C8	107.7 (2)
C10—Fe1—C5	106.98 (10)	C6—C7—Fe1	69.19 (13)
C2—Fe1—C5	68.83 (8)	C8—C7—Fe1	69.54 (12)
C1—Fe1—C6	106.79 (10)	C6—C7—H7	126.1
C10—Fe1—C6	40.44 (10)	C8—C7—H7	126.1
C2—Fe1—C6	122.59 (9)	Fe1—C7—H7	126.7
C5—Fe1—C6	122.90 (10)	C9—C8—C7	107.8 (2)
C1—Fe1—C9	157.79 (9)	C9—C8—Fe1	69.58 (12)
C10—Fe1—C9	40.52 (9)	C7—C8—Fe1	69.93 (12)
C2—Fe1—C9	159.87 (9)	C9—C8—H8	126.1
C5—Fe1—C9	121.99 (9)	C7—C8—H8	126.1
C6—Fe1—C9	68.08 (9)	Fe1—C8—H8	126.0
C1—Fe1—C8	159.80 (8)	C8—C9—C10	108.1 (2)
C10—Fe1—C8	68.07 (10)	C8—C9—Fe1	70.04 (12)
C2—Fe1—C8	123.80 (9)	C10—C9—Fe1	69.47 (12)
C5—Fe1—C8	158.01 (9)	C8—C9—H9	126.0
C6—Fe1—C8	68.15 (10)	C10—C9—H9	126.0
C9—Fe1—C8	40.38 (9)	Fe1—C9—H9	126.1
C1—Fe1—C4	68.90 (9)	C6—C10—C9	108.2 (2)
C10—Fe1—C4	123.05 (10)	C6—C10—Fe1	69.89 (12)
C2—Fe1—C4	68.52 (9)	C9—C10—Fe1	70.01 (12)
C5—Fe1—C4	40.47 (10)	C6—C10—H10	125.9
C6—Fe1—C4	159.02 (9)	C9—C10—H10	125.9
C9—Fe1—C4	107.71 (9)	Fe1—C10—H10	125.8
C8—Fe1—C4	122.89 (10)	C1—C11—N1	111.13 (16)
C1—Fe1—C3	68.67 (10)	C1—C11—H11A	109.4
C10—Fe1—C3	159.57 (9)	N1—C11—H11A	109.4
C2—Fe1—C3	40.55 (9)	C1—C11—H11B	109.4
C5—Fe1—C3	68.13 (10)	N1—C11—H11B	109.4
C6—Fe1—C3	158.90 (9)	H11A—C11—H11B	108.0
C9—Fe1—C3	123.88 (9)	N1—C12—C13	112.49 (16)
C8—Fe1—C3	108.60 (10)	N1—C12—C14	107.53 (15)
C4—Fe1—C3	40.47 (9)	C13—C12—C14	113.21 (17)
C1—Fe1—C7	123.06 (9)	N1—C12—H12	107.8
C10—Fe1—C7	67.96 (10)	C13—C12—H12	107.8
C2—Fe1—C7	108.04 (10)	C14—C12—H12	107.8
C5—Fe1—C7	159.48 (9)	O1—C13—C12	114.61 (17)
C6—Fe1—C7	40.43 (10)	O1—C13—H13A	108.6
C9—Fe1—C7	67.97 (9)	C12—C13—H13A	108.6
C8—Fe1—C7	40.53 (10)	O1—C13—H13B	108.6
C4—Fe1—C7	158.99 (9)	C12—C13—H13B	108.6
C3—Fe1—C7	123.54 (10)	H13A—C13—H13B	107.6
C11—N1—C12	114.96 (15)	C12—C14—C15	111.95 (17)
C11—N1—H91	106.0	C15—C14—H14A	109.2
C12—N1—H91	111.0	C12—C14—H14A	109.2
C11—N1—H92	105.9	C15—C14—H14B	109.2
C12—N1—H92	109.2	C12—C14—H14B	109.2
H91—N1—H92	109.5	H14A—C14—H14B	107.9
C13—O1—H93	116.1	C16—C15—C20	118.7 (2)
C2—C1—C5	107.5 (2)	C16—C15—C14	120.54 (19)
C2—C1—C11	127.15 (18)	C20—C15—C14	120.7 (2)
C5—C1—C11	125.4 (2)	C15—C16—C17	120.7 (2)
C2—C1—Fe1	69.96 (11)	C15—C16—H16	119.6
C5—C1—Fe1	69.98 (9)	C17—C16—H16	119.6
C11—C1—Fe1	125.48 (16)	C16—C17—C18	120.2 (2)
C3—C2—C1	107.84 (18)	C16—C17—H17	119.9
C3—C2—Fe1	70.21 (12)	C18—C17—H17	119.9
C1—C2—Fe1	68.91 (11)	C19—C18—C17	119.5 (2)
C3—C2—H2	126.1	C19—C18—H18	120.3
C1—C2—H2	126.1	C17—C18—H18	120.3
Fe1—C2—H2	126.4	C18—C19—C20	119.9 (2)
C2—C3—C4	108.53 (18)	C18—C19—H19	120.0
C2—C3—Fe1	69.24 (12)	C20—C19—H19	120.0
C4—C3—Fe1	69.75 (12)	C15—C20—C19	120.9 (2)
C2—C3—H3	125.7	C15—C20—H20	119.6
C4—C3—H3	125.7	C19—C20—H20	119.6
Fe1—C3—H3	126.9	O2—C21—O3	123.9 (2)
C5—C4—C3	107.96 (19)	O2—C21—C22	116.6 (2)
C5—C4—Fe1	69.37 (12)	O3—C21—C22	119.5 (2)
C3—C4—Fe1	69.79 (12)	C23—C22—C21	125.2 (2)
C5—C4—H4	126.0	C23—C22—H22	117.4
C3—C4—H4	126.0	C21—C22—H22	117.4
Fe1—C4—H4	126.4	C22—C23—C24	128.1 (3)
C4—C5—C1	108.2 (2)	C22—C23—H23	115.9
C4—C5—Fe1	70.16 (11)	C24—C23—H23	115.9
C1—C5—Fe1	68.79 (10)	C23—C24—H24A	109.5
C4—C5—H5	125.9	C23—C24—H24B	109.5
C1—C5—H5	125.9	H24A—C24—H24B	109.5
Fe1—C5—H5	126.7	C23—C24—H24C	109.5
C10—C6—C7	108.2 (2)	H24A—C24—H24C	109.5
C10—C6—Fe1	69.67 (13)	H24B—C24—H24C	109.5
C7—C6—Fe1	70.37 (13)		
			
C10—Fe1—C1—C2	162.25 (13)	C4—Fe1—C6—C10	44.6 (3)
C5—Fe1—C1—C2	−118.3 (2)	C3—Fe1—C6—C10	−166.5 (2)
C6—Fe1—C1—C2	120.65 (14)	C7—Fe1—C6—C10	−119.01 (19)
C9—Fe1—C1—C2	−166.4 (2)	C1—Fe1—C6—C7	−121.63 (14)
C8—Fe1—C1—C2	48.5 (4)	C10—Fe1—C6—C7	119.01 (19)
C4—Fe1—C1—C2	−81.09 (14)	C2—Fe1—C6—C7	−79.43 (15)
C3—Fe1—C1—C2	−37.53 (13)	C5—Fe1—C6—C7	−163.87 (13)
C7—Fe1—C1—C2	79.43 (17)	C9—Fe1—C6—C7	81.30 (15)
C10—Fe1—C1—C5	−79.49 (18)	C8—Fe1—C6—C7	37.63 (13)
C2—Fe1—C1—C5	118.3 (2)	C4—Fe1—C6—C7	163.6 (2)
C6—Fe1—C1—C5	−121.09 (16)	C3—Fe1—C6—C7	−47.5 (3)
C9—Fe1—C1—C5	−48.1 (3)	C10—C6—C7—C8	0.6 (2)
C8—Fe1—C1—C5	166.7 (3)	Fe1—C6—C7—C8	−59.06 (16)
C4—Fe1—C1—C5	37.17 (15)	C10—C6—C7—Fe1	59.66 (15)
C3—Fe1—C1—C5	80.73 (16)	C1—Fe1—C7—C6	76.56 (17)
C7—Fe1—C1—C5	−162.31 (14)	C10—Fe1—C7—C6	−37.74 (14)
C10—Fe1—C1—C11	40.3 (2)	C2—Fe1—C7—C6	119.41 (14)
C2—Fe1—C1—C11	−122.0 (2)	C5—Fe1—C7—C6	41.7 (3)
C5—Fe1—C1—C11	119.8 (3)	C9—Fe1—C7—C6	−81.61 (14)
C6—Fe1—C1—C11	−1.3 (2)	C8—Fe1—C7—C6	−119.3 (2)
C9—Fe1—C1—C11	71.7 (3)	C4—Fe1—C7—C6	−163.7 (2)
C8—Fe1—C1—C11	−73.5 (4)	C3—Fe1—C7—C6	161.43 (13)
C4—Fe1—C1—C11	156.9 (2)	C1—Fe1—C7—C8	−164.13 (15)
C3—Fe1—C1—C11	−159.50 (19)	C10—Fe1—C7—C8	81.57 (16)
C7—Fe1—C1—C11	−42.5 (2)	C2—Fe1—C7—C8	−121.28 (15)
C5—C1—C2—C3	−0.5 (2)	C5—Fe1—C7—C8	161.0 (2)
C11—C1—C2—C3	179.57 (19)	C6—Fe1—C7—C8	119.3 (2)
Fe1—C1—C2—C3	59.65 (15)	C9—Fe1—C7—C8	37.70 (14)
C5—C1—C2—Fe1	−60.19 (13)	C4—Fe1—C7—C8	−44.4 (3)
C11—C1—C2—Fe1	119.9 (2)	C3—Fe1—C7—C8	−79.26 (17)
C1—Fe1—C2—C3	−119.19 (18)	C6—C7—C8—C9	−0.6 (2)
C10—Fe1—C2—C3	−163.5 (2)	Fe1—C7—C8—C9	−59.48 (14)
C5—Fe1—C2—C3	−80.69 (14)	C6—C7—C8—Fe1	58.85 (15)
C6—Fe1—C2—C3	162.97 (13)	C1—Fe1—C8—C9	160.5 (3)
C9—Fe1—C2—C3	45.8 (3)	C10—Fe1—C8—C9	37.68 (14)
C8—Fe1—C2—C3	78.92 (16)	C2—Fe1—C8—C9	−163.12 (13)
C4—Fe1—C2—C3	−37.10 (13)	C5—Fe1—C8—C9	−43.3 (4)
C7—Fe1—C2—C3	120.86 (14)	C6—Fe1—C8—C9	81.42 (15)
C10—Fe1—C2—C1	−44.3 (3)	C4—Fe1—C8—C9	−78.40 (17)
C5—Fe1—C2—C1	38.51 (13)	C3—Fe1—C8—C9	−120.82 (14)
C6—Fe1—C2—C1	−77.83 (15)	C7—Fe1—C8—C9	119.0 (2)
C9—Fe1—C2—C1	165.0 (2)	C1—Fe1—C8—C7	41.6 (4)
C8—Fe1—C2—C1	−161.88 (13)	C10—Fe1—C8—C7	−81.28 (16)
C4—Fe1—C2—C1	82.10 (13)	C2—Fe1—C8—C7	77.92 (18)
C3—Fe1—C2—C1	119.19 (18)	C5—Fe1—C8—C7	−162.3 (3)
C7—Fe1—C2—C1	−119.94 (13)	C6—Fe1—C8—C7	−37.54 (15)
C1—C2—C3—C4	0.0 (2)	C9—Fe1—C8—C7	−119.0 (2)
Fe1—C2—C3—C4	58.83 (15)	C4—Fe1—C8—C7	162.64 (14)
C1—C2—C3—Fe1	−58.83 (14)	C3—Fe1—C8—C7	120.22 (15)
C1—Fe1—C3—C2	38.06 (12)	C7—C8—C9—C10	0.4 (2)
C10—Fe1—C3—C2	162.4 (2)	Fe1—C8—C9—C10	−59.28 (15)
C5—Fe1—C3—C2	82.56 (13)	C7—C8—C9—Fe1	59.70 (15)
C6—Fe1—C3—C2	−43.3 (3)	C1—Fe1—C9—C8	−162.3 (2)
C9—Fe1—C3—C2	−162.72 (13)	C10—Fe1—C9—C8	−119.2 (2)
C8—Fe1—C3—C2	−120.63 (13)	C2—Fe1—C9—C8	44.5 (3)
C4—Fe1—C3—C2	120.14 (18)	C5—Fe1—C9—C8	162.37 (16)
C7—Fe1—C3—C2	−78.28 (15)	C6—Fe1—C9—C8	−81.59 (16)
C1—Fe1—C3—C4	−82.08 (13)	C4—Fe1—C9—C8	120.28 (15)
C10—Fe1—C3—C4	42.3 (3)	C3—Fe1—C9—C8	78.66 (17)
C2—Fe1—C3—C4	−120.14 (18)	C7—Fe1—C9—C8	−37.84 (15)
C5—Fe1—C3—C4	−37.58 (12)	C1—Fe1—C9—C10	−43.1 (3)
C6—Fe1—C3—C4	−163.4 (2)	C2—Fe1—C9—C10	163.7 (2)
C9—Fe1—C3—C4	77.14 (15)	C5—Fe1—C9—C10	−78.40 (16)
C8—Fe1—C3—C4	119.22 (13)	C6—Fe1—C9—C10	37.63 (14)
C7—Fe1—C3—C4	161.58 (13)	C8—Fe1—C9—C10	119.2 (2)
C2—C3—C4—C5	0.5 (2)	C4—Fe1—C9—C10	−120.49 (14)
Fe1—C3—C4—C5	59.07 (15)	C3—Fe1—C9—C10	−162.11 (14)
C2—C3—C4—Fe1	−58.52 (15)	C7—Fe1—C9—C10	81.39 (15)
C1—Fe1—C4—C5	−37.85 (13)	C7—C6—C10—C9	−0.3 (2)
C10—Fe1—C4—C5	76.95 (15)	Fe1—C6—C10—C9	59.76 (15)
C2—Fe1—C4—C5	−82.15 (13)	C7—C6—C10—Fe1	−60.10 (15)
C6—Fe1—C4—C5	44.0 (3)	C8—C9—C10—C6	−0.1 (2)
C9—Fe1—C4—C5	118.85 (13)	Fe1—C9—C10—C6	−59.68 (15)
C8—Fe1—C4—C5	160.63 (13)	C8—C9—C10—Fe1	59.63 (15)
C3—Fe1—C4—C5	−119.32 (18)	C1—Fe1—C10—C6	−78.49 (15)
C7—Fe1—C4—C5	−166.6 (2)	C2—Fe1—C10—C6	−45.8 (3)
C1—Fe1—C4—C3	81.46 (14)	C5—Fe1—C10—C6	−121.15 (14)
C10—Fe1—C4—C3	−163.74 (12)	C9—Fe1—C10—C6	119.15 (19)
C2—Fe1—C4—C3	37.17 (12)	C8—Fe1—C10—C6	81.60 (15)
C5—Fe1—C4—C3	119.32 (18)	C4—Fe1—C10—C6	−162.53 (13)
C6—Fe1—C4—C3	163.3 (2)	C3—Fe1—C10—C6	166.1 (2)
C9—Fe1—C4—C3	−121.83 (13)	C7—Fe1—C10—C6	37.73 (14)
C8—Fe1—C4—C3	−80.05 (15)	C1—Fe1—C10—C9	162.36 (13)
C7—Fe1—C4—C3	−47.3 (3)	C2—Fe1—C10—C9	−165.0 (2)
C3—C4—C5—C1	−0.9 (2)	C5—Fe1—C10—C9	119.70 (14)
Fe1—C4—C5—C1	58.45 (14)	C6—Fe1—C10—C9	−119.15 (19)
C3—C4—C5—Fe1	−59.33 (15)	C8—Fe1—C10—C9	−37.56 (14)
C2—C1—C5—C4	0.9 (2)	C4—Fe1—C10—C9	78.31 (16)
C11—C1—C5—C4	−179.22 (19)	C3—Fe1—C10—C9	46.9 (3)
Fe1—C1—C5—C4	−59.30 (14)	C7—Fe1—C10—C9	−81.43 (15)
C2—C1—C5—Fe1	60.18 (13)	C2—C1—C11—N1	91.1 (2)
C11—C1—C5—Fe1	−119.9 (2)	C5—C1—C11—N1	−88.8 (2)
C1—Fe1—C5—C4	119.7 (2)	Fe1—C1—C11—N1	−178.21 (13)
C10—Fe1—C5—C4	−121.38 (14)	C12—N1—C11—C1	163.62 (17)
C2—Fe1—C5—C4	81.30 (14)	C11—N1—C12—C13	47.3 (2)
C6—Fe1—C5—C4	−162.76 (13)	C11—N1—C12—C14	172.66 (17)
C9—Fe1—C5—C4	−79.66 (16)	N1—C12—C13—O1	68.3 (2)
C8—Fe1—C5—C4	−48.1 (3)	C14—C12—C13—O1	−53.8 (2)
C3—Fe1—C5—C4	37.57 (13)	N1—C12—C14—C15	−179.54 (17)
C7—Fe1—C5—C4	166.3 (2)	C13—C12—C14—C15	−54.7 (2)
C10—Fe1—C5—C1	118.91 (16)	C12—C14—C15—C16	−78.0 (2)
C2—Fe1—C5—C1	−38.41 (15)	C12—C14—C15—C20	101.5 (2)
C6—Fe1—C5—C1	77.52 (17)	C20—C15—C16—C17	−0.4 (3)
C9—Fe1—C5—C1	160.63 (15)	C14—C15—C16—C17	179.08 (19)
C8—Fe1—C5—C1	−167.8 (3)	C15—C16—C17—C18	1.0 (3)
C4—Fe1—C5—C1	−119.7 (2)	C16—C17—C18—C19	−0.7 (3)
C3—Fe1—C5—C1	−82.14 (16)	C17—C18—C19—C20	−0.1 (4)
C7—Fe1—C5—C1	46.6 (3)	C16—C15—C20—C19	−0.4 (3)
C1—Fe1—C6—C10	119.37 (13)	C14—C15—C20—C19	−179.93 (19)
C2—Fe1—C6—C10	161.56 (13)	C18—C19—C20—C15	0.7 (4)
C5—Fe1—C6—C10	77.12 (15)	O2—C21—C22—C23	−149.0 (3)
C9—Fe1—C6—C10	−37.71 (13)	O3—C21—C22—C23	29.3 (4)
C8—Fe1—C6—C10	−81.38 (14)	C21—C22—C23—C24	−179.6 (3)

### Hydrogen-bond geometry (Å, °)

**Table:**

*D*—H···*A*	*D*—H	H···*A*	*D*···*A*	*D*—H···*A*
N1—H91···O2^i^	0.95	1.74	2.685 (2)	173
N1—H92···O3	0.82	1.94	2.747 (2)	170
O1—H93···O3^i^	0.87	1.85	2.712 (2)	172
C16—H16···O1^ii^	0.93	2.56	3.447 (3)	159
C18—H18···O2^iii^	0.93	2.58	3.435 (3)	154

Symmetry codes: (i) *x*−1, *y*, *z*;  (ii) *x*+1, *y*, *z*; (iii) −*x*+1, *y*−1/2, −*z*+1.

## References

[bb1] Altomare, A., Burla, M. C., Camalli, M., Cascarano, G. L., Giacovazzo, C., Guagliardi, A., Moliterni, A. G. G., Polidori, G. & Spagna, R. (1999). *J. Appl. Cryst.* **32**, 115–119.

[bb2] Braga, D., Curzi, M., Giaffreda, S. L., Grepioni, F., Maini, L., Pettersen, A. & Polito, M. (2008). *Ferrocenes: Ligands, Materials and Biomolecules*, edited by P. Štěpnička, pp. 465–498, Chichester: Wiley.

[bb3] Flack, H. D. (1983). *Acta Cryst.* A**39**, 876–881.

[bb4] Hess, A., Brosch, O., Weyhermüller, T. & Metzler-Nolte, N. (1999). *J. Organomet. Chem.* **589**, 75–84.

[bb5] Nonius (2000). *COLLECT* Nonius BV, Delft, The Netherlands.

[bb6] Otwinowski, Z. & Minor, W. (1997). *Methods in Enzymology*, Vol. 276, *Macromolecular Crystallography*, Part A, edited by C. W. Carter Jr & R. M. Sweet, pp. 307–326. New York: Academic Press.

[bb7] Sheldrick, G. M. (2008). *Acta Cryst.* A**64**, 112–122. 10.1107/S0108767307043930 18156677

[bb8] Spek, A. L. (2009). *Acta Cryst.* D**65**, 148–155. 10.1107/S090744490804362X PMC2631630 19171970

[bb9] Štěpnička, P., Císařová, I. & Ludvík, J. (2004). *J. Organomet. Chem.* **689**, 631–638.

[bb10] Štěpnička, P., Zábranský, M., Císařová, I. & Lamač, M. (2008*a*). *J. Organomet. Chem.* **693**, 3831–3841.

[bb11] Štěpnička, P., Zábranský, M., Lamač, M., Císařová, I. & Němec, P. (2008*b*). *J. Organomet. Chem.* **693**, 1779–1786.

